# Influences of a Supplemental Blend of Essential Oils Plus 25-Hydroxy-Vit-D3 and Zilpaterol Hydrochloride (β2 Agonist) on Growth Performance and Carcass Measures of Feedlot Lambs Finished under Conditions of High Ambient Temperature

**DOI:** 10.3390/ani14091391

**Published:** 2024-05-06

**Authors:** Alfredo Estrada-Angulo, Moisés Verdugo-Insúa, Lucía de G. Escobedo-Gallegos, Beatriz I. Castro-Pérez, Jesús D. Urías-Estrada, Elizama Ponce-Barraza, Daniel Mendoza-Cortez, Francisco G. Ríos-Rincón, Francisco Monge-Navarro, Alberto Barreras, Richard A. Zinn, Luis Corona-Gochi, Alejandro Plascencia

**Affiliations:** 1 Faculty of Veterinary Medicine and Zootechnics, Autonomous University of Sinaloa, Culiacan 80260, Sinaloa, Mexico; alfred_vet@hotmail.com (A.E.-A.); mvz.verdugo.4019@gmail.com (M.V.-I.); lucia.escobedo@uabc.edu.mx (L.d.G.E.-G.); laisa_29@hotmail.com (B.I.C.-P.); david.urias@uas.edu.mx (J.D.U.-E.); elizama.ponce@hotmail.com (E.P.-B.); danielmendoza@outlook.com (D.M.-C.); fgrios@uas.edu.mx (F.G.R.-R.); 2 Veterinary Science Research Institute, Autonomous University of Baja California, Mexicali 21100, Baja California, Mexico; fmongenavarro@gmail.com (F.M.-N.); beto_barreras@yahoo.com (A.B.); 3 Animal Science Department, University of California, Davis, CA 95616, USA; razinn@ucdavis.edu; 4 Faculty of Veterinary Medicine and Zootechnics, National Autonomous University of Mexico, Mexico City 04510, Mexico; gochi@unam.mx

**Keywords:** essential oils, zilpaterol chlorhydrate, feedlot lambs, growth performance, energetics, carcass

## Abstract

**Simple Summary:**

Combining feed additives is a common practice in the livestock feed industry. Of interest in this study is whether growth performance responses to the combination of essential oils enriched with vitamin D3 (EOD3) with the beta-agonist zilpaterol hydrochloride (ZH) will be additive. Based on our results, growth performance responses to the combination of EOD3 with ZH are additive. In addition, ZH enhanced carcass traits.

**Abstract:**

Forty-eight Pelibuey × Katahdin male intact lambs (25.12 ± 3.79 kg LW) were used in a 70-d growing-finishing trial. Dietary treatments consisted of total mixed corn-based diet supplemented with: (1) no feed additives (Control); (2) 150 mg of essential oils blend plus 0.10 mg of 25-hydroxy-Vit-D3/kg diet offered throughout the 70-d experimental period (EOD3); (3) Control diet fed during the first 35 days and zilpaterol hydrochloride (ZH) supplementation at 6 mg/kg diet offered during the final 35 days of the experiment (32 days with ZH with a withdrawal 3-d before harvest), and (4) basal diet supplemented with EOD3 during first 35 days finishing, and EOD3 in combination with ZH (EOD3 + ZH) during the subsequent 32-days with ZH withdrawal 3 days before harvest. The temperature–humidity index during the experiment averaged 80.4 ± 3.2. There were no treatment interactions (*p* > 0.20) on growth performance and carcass measures. Supplemental EOD3 did not affect (*p* = 0.43) dry matter intake (DMI), but increased (*p* < 0.01) carcass adjusted average daily gain (ADG, 9.2%), gain efficiency (GF, 6.7%), and observed vs. expected dietary net energy for maintenance (NE_m_, 4.8%) and for gain (NE_g_, 6.4%). Supplemental ZH did not affect dry matter intake (DMI, *p* = 0.50) but increased (*p* < 0.01) carcass adjusted ADG (14.5%), GF (13%) and observed vs. expected dietary NE_m_ (9%) and NE_g_ (11.7%). Compared to control lambs, the combination of both additives increased ADG (24.9%), GF (21.2%), and observed vs. expected dietary NE_m_ and NE_g_ (14.2% and 18.9%, respectively). There were no treatment interactions on carcass characteristics, visceral organ mass, or on gene expression of IGF1, IGF2 and mTOR in longissimus muscle (LM). Supplemental EOD3 increased hot carcass weight (HCW; 4.0%, *p* < 0.01) but did not affect other carcass measures. Supplemental EOD3 decreased (3%, *p* = 0.03) intestine mass weight (g intestine/kg empty body weight). Supplemental ZH increased HCW (6%, *p* < 0.01), dressing percentage (1.7%, *p* = 0.04), and LM area (9.7%, *p* < 0.01), and decreased kidney-pelvic-fat percentage (16.2%, *p* < 0.01), fat thickness (14.7%, *p* = 0.03), and visceral fat. Compared to controls, the combination of EOD3 with ZH increased HCW (10.2%). It is concluded that growth performance responses to supplemental EOD3 and ZH are additive. Both supplements can be fed in combination without detrimental effects on expected benefits when fed separately. In addition, ZH supplementation improves carcass traits.

## 1. Introduction

Globally, a significant proportion of feedlot lambs are finished in semi-arid, tropical, and subtropical climates [1]. These regions have high ambient heat load (HAHL) during a major portion of the year. Conditions of HAHL result in reduced energy intake and growth performance, particularly during the finishing phase [2]. Reduced feed intake, a typical phenomenon observed during HAHL, is a primary driver for reductions in ADG and rate of muscle accretion [3]. However, accumulating evidence indicates that HAHL negatively influences not only feed intake (and therefore, energy intake), but efficiency of energy utilization of the diet [4]. Several management strategies (shade, sprinklers, overhead fans, among others) have been tested to mitigate the negative effects of HAHL on rate of gain, energy efficiency and carcass characteristics in finishing cattle [5,6]. Additionally, feed additives may play an important role in amelioration of negative effects of HAHL on productivity. Zilpaterol hydrochloride (ZH), a β2 adrenergic agonist extensively used as feed additive in finishing diets for feedlot cattle in several countries [7], stimulates muscle growth even when lambs were finished under HAHL conditions [8]. Likewise, the use of blends of essential oils as feed additives has gained popularity as a strategy to decrease the negative impact of HAHL on ADG and gain efficiency [9,10] but without improvements on the carcass. Recently, it has been reported that supra-supplementation of 25-hydroxy-vitamin-D3 (dosed from 0.003 to 0.006 mg/kg LW) enhanced carcass measures of feedlot cattle [11,12]. Mendoza-Cortéz et al. [13] performed an experiment to compare the combination of a blend essential oils (119 mg/kg of diet) plus 0.12 mg 25-hydroxy-vitamin-D3/kg/of diet (EOD3) vs. the ionophore monensin (24 mg/kg diet) in growing-finishing cattle under HAHL conditions. Compared with supplemental monensin, the EOD3 combination enhanced ADG and gain efficiency, and decreased (7.7%) the estimated maintenance coefficient (MQ). It was hypothesized that the reduction in MQ, and hence the enhanced energy efficiency, of cattle supplemented with EOD3 under HAHL conditions, might be the basis for increased net protein retention. Likewise, Escobedo-Gallegos et al. [14] observed that, compared to non-supplemented controls, supplemental monensin and supplemental EOD3 increased (4.4%) dietary energetic efficiency and carcass characteristics of lambs finished under HAHL conditions. However, the interaction of supplemental EOD3 when fed in combination with ZH has not been evaluated. Consequently, the objective of this study was to evaluated the main effects and interactions of EOD3 and zilpaterol hydrochloride (β2 agonist) on growth performance, dietary energetics, carcass characteristics, and visceral mass of finishing lambs fed under HAHL conditions. In addition, potential treatment effects on muscle expression of mRNA for IGF1, IGF2, and mTOR were also evaluated.

## 2. Material and Methods

### 2.1. Location of the Study

The experiment was conducted at the Universidad Autónoma de Sinaloa Feedlot Lamb Research Unit located in Culiacán, México (24°46′13″ N and 107°21′14″ W). Culiacán is about 55 m above sea level and has a tropical climate. During the experiment, ambient air temperature averaged 29.2 °C (minimum and maximum of 28.8 °C and 29.60 °C, respectively), and relative humidity averaged 73.1% (minimum and maximum of 71.4% and 74.8%, respectively). All animal management procedures were conducted within the guidelines of federally and locally approved techniques for animal use and care [15] and approved by the Ethics Committee of the Faculty of Veterinary Medicine and Zootechnics of the Autonomous University of Sinaloa (Protocol #04292023).

### 2.2. Climatic Variables and Temperature Humidity Index (THI) Calculation

Climatic variables (ambient temperature and relative humidity) were obtained every hour from two on-site weather stations (Thermo-hygrometer Avaly, Mod. DTH880, Mofeg S.A., Zapopan, Jalisco, Mexico). The temperature humidity index (THI) was calculated using the following formula: THI = 0.81 × T + (RH/100) × (T − 14.40) + 46.40, where T = temperature expressed in Celsius grade and RH = relative humidity [16].

### 2.3. Animals, Diet, and Experimental Design

**Figure 1:**
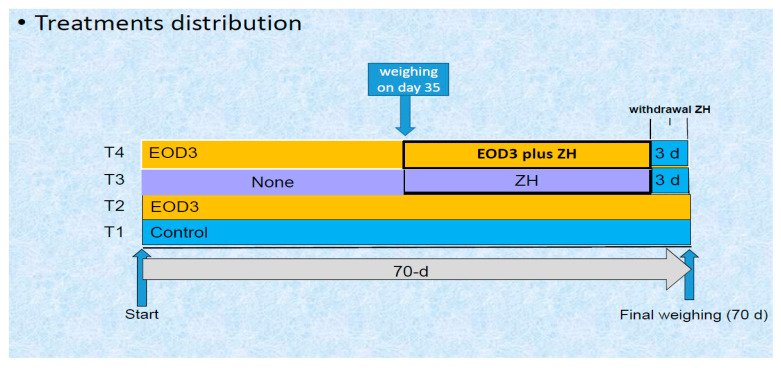
Treatment distribution: Control = basal diet without feed additives; EOD3 = 150 mg of essential oils blend plus 0.09 mg of 25-hydroxy- Vit-D3/kg of diet offered during all experimental period; ZH = basal diet during the first 35 days and zilpaterol hydrochloride supplementation at 6.0 mg/kg of diet offered during the final 35 days of the experiment (33 days with ZH with a withdrawal 3-d at moment of harvest, and EOD3 + ZH = basal diet supplemented with EOD3 during first 35 days and finishing with the combination with ZH (**EOD3 + ZH**) during the final 35 days of the experiment with a 3-d withdrawal from ZH before harvest).

Forty-eight Pelibuey × Katahdin male intact lambs (176 ± 18 d age; 25.1 ± 3.8 kg LW) were used to evaluate the effects of feeding with a blend of essential oils plus 25-hydroxy-Vit-D3 (EOD3), zilpaterol hydrochloride (ZH) or their combination (EOD3 + ZH) on growth performance, dietary energetics, carcass characteristics, visceral mass, and gene expression of mRNA for IGF1, IGF2, and mTOR in longissimus muscle. Three weeks before the initiation of the experiment, all lambs were treated for parasites (Albendaphorte 10%, Animal Health and Welfare, México City, México), injected with 1 × 10^6^ IU vitamin A (Synt-ADE^®^, Fort Dodge, Animal Health, México City, Mexico), and vaccinated for *Mannheimia haemolityca* (One shot Pfizer, México City, Mexico). All lambs were adapted to the basal finishing diet (Table 1) and facilities 21 day before the start of the experiment. The basal diet was prepared using a 2.5 m^3^ capacity paddle mixer (model 30910-7, Coyoacán, México). Feed samples (~50 g) from each batch were taken and stored (4 °C) in sealed bags. Upon initiation of the experiment, all lambs (n = 48) were weighed before the morning meal (electronic scale; TORREY TIL/S: 107 2691, TOR REY Electronics Inc., Houston, TX, USA), and assigned within six weight groupings to 24 pens, with two lambs per pen (6 replicates per treatment). Pens were 6 m^2^ with overhead shade, automatic waterers and 1 m fence-line feed bunks. The dosage level of supplemental EO was based on prior studies, in which positive responses in growth performance of finishing lambs were observed at 150 mg EO/kg of diet [9,10]. The dosage level of D3 was based on positive responses in growth performance and carcass in lambs [14] and feedlot cattle [11,12], where levels of supplementation ranged between 0.002 and 0.006 mg D3/kg LW. Therefore, based on initial weight, feed intake, and average live weight for Katahdin × Pelibuey lambs observed in previous growing-finishing experiments [17,18,19], a dietary concentration of 0.10 mg D3/kg was selected to provide an average of 0.003 mg D3/kg LW. The dosage of ZH (6 mg/kg diet) has proved optimal to improve growth performance and carcass characteristics for finishing lambs [20]. Therefore, dietary treatments consisted of total mixed corn-based diet (Table 1) supplemented with: (1) no feed additives (Control); (2) 150 mg of essential oils blend plus 0.10 mg of 25-hydroxy-Vit-D3/kg diet offered throughout the 70-d experimental period (EOD3); (3) Control diet fed during the first 35 days and zilpaterol hydrochloride (ZH) supplementation at 6 mg/kg diet offered during the final 35 days of the experiment (32 days with ZH with a withdrawal 3-d before harvest), and (4) basal diet supplemented with EOD3 during first 35 days finishing, and EOD3 in combination with ZH (EOD3 + ZH) during the subsequent 32-days, with ZH withdrawal 3 days before harvest. Treatment design is shown in Figure 1. The experiment lasted 70 days. The sources of the blended oils (EO) and 25-hydroxy-Vit-D3 (D3) used were the products CRINA-Ruminant^®^ and Hy-D^®^ (DSM Nutritional Products, Basel, Switzerland). CRINA-Ruminant^®^ contains a standardized mixture of essential oils, including thymol, eugenol, vanillin, guaiac, and limonene. Source of ZH was Zilmax^®^ (MSD Salud Animal México, Santiago Tianguistenco, México). The doses of respective treatments were previously weighed and diluted with ground rice husk using a micro blender powder mixer (TRITTON; Cylindrical type mixer Mod 200 L, Guadalajara, JL, Mexico). To ensure the concentration of the planned dosages, the prepared dilution of each supplement was hand-mixed with 30 g of wheat bran and provided with the morning feed. Fresh feed was provided twice daily at 0800 and 1400 h in a 40:60 proportion of the total daily feed consumption (as-fed basis). Whereas the amount of feed provided in the morning feeding was constant, feed offered in the afternoon feeding was adjusted daily, allowing for a feed residual ~50 g/kg daily feed offering. Residual feed of each pen was collected between 0740 and 0750 h each morning and weighed to determine the feed intake. Upon initiation of the study, lambs were individually weighed prior to the morning feeding (0730 h). Initial live weight (LW) was converted to shrunk body weight (SBW) by multiplying LW by 0.96 to adjust for the gastro-intestinal fill [21]. All lambs were fasted (from feed, but not for drinking water) for 18 h before individual weighing to determine final fasted LW. Feed samples were collected from each elaborated batch. Feed refusals were collected daily and composited weekly for DM analysis (oven drying at 105 °C until no further weight loss; method 930.15) [22].

### 2.4. Chemical Analysis

Feed samples were subjected to the following analyses: DM (oven drying at 105 °C until no further weight loss; method 930.15) and CP (N × 6.25, method 984.13) according to AOAC [22]. Neutral detergent fiber (NDF) was determined following procedures described by Van Soest et al. [24] (corrected for NDF-ash, incorporating heat stable α-amylase using Ankom Technology, Macedon, NY, USA).

### 2.5. Calculations

Estimates of ADG and dietary net energy are based on initial SBW and final (d 70) fasted SBW. Because there was a significant difference in carcass dressing percentage between the treatments, final SBW was adjusted for hot carcass weight (HCW) by dividing individual HCW by the average dressing percentage (0.5813) for all lambs. The average daily gain was computed by subtracting the initial SBW from final SBW and dividing the result by the number of days on feed. Gain efficiency was computed as ADG/average DMI. One approach for evaluation of the efficiency of dietary energy utilization in growth–performance trials is the ratio of observed-to-expected DMI and observed-to-expected dietary net energy (NE). Based on estimated diet NE concentration and measures of growth performance, there is an expected energy intake. This estimation of expected DMI is performed based on observed ADG, average SBW, and NE values of the diet (Table 1): expected DMI, kg/d = (EM/2.10) + (EG/1.44), where EM (energy required for maintenance, Mcal/d) = 0.056 × SBW^0.75^, EG (energy gain, Mcal/d) = 0.276 × ADG × SBW^0.75^, and 2.10 and 1.44 are the NE_m_ and NE_g_ values contained in the basal diet according to the tabular values from NRC [23]. The coefficient (0.276) was taken from NRC [25], assuming a mature weight of 113 kg for Pelibuey × Katahdin male lambs [26]. The observed dietary NE was calculated using EM and EG values and the DMI observed during the experiment by means of the quadratic formula:

$$x=\frac{-b\pm \sqrt{b^{2}-4ac}}{2c}$$

where *x* = observed dietary NE_m_, Mcal/kg, *a* = −0.41 EM, *b* = 0.877 EM + 0.41 DMI + EG, and *c* = −0.877 DMI [27].

### 2.6. Carcass Characteristics and Visceral Mass Data

All the lambs were harvested on the same day. After sacrifice, lambs were skinned, and the gastrointestinal organs were separated and weighed. After carcasses (with kidneys and internal fat included) were chilled in a cooler at −2 to 1 °C for 24 h, the following measurements were obtained: (1) body wall thickness (at a point between the 12th and 13th rib, five inches from the midline of the carcass); (2) fat thickness perpendicular to the *m. longissimus thoracis* (LM), measured over the center of the ribeye between the 12th and 13th rib; (3) LM surface area, measured using a grid reading of the cross-sectional area of the ribeye between the 12th and 13th rib; and (4) kidney, pelvic, and heart fat (KPH). The KPH was manually removed from the carcass, weighed, and reported as a percentage of the cold carcass weight (CCW) according to USDA [28]. Components of the gastro-intestinal tract (GIT), including the tongue, esophagus, stomach (rumen, reticulum, omasum, and abomasum), pancreas, liver, gallbladder, small intestine (duodenum, jejunum, and ileum), and large intestine (caecum, colon, and rectum), were removed and weighed. The GIT was then washed, drained, and weighed to obtain empty weights. The difference between full and washed digesta-free GIT was subtracted from the SBW to determine the empty body weight (EBW). All tissue weights are reported on a fresh tissue basis. Organ mass is expressed as grams of fresh tissue per kilogram of final EBW, where final EBW represents the final full live weight minus the total digesta weight. The full visceral mass was calculated by the summation of all visceral components (stomach complex + small intestine + large intestine + liver + lungs + heart), including digesta. The stomach complex was calculated as the digesta-free sum of the weights of the rumen, reticulum, omasum, and abomasum.

### 2.7. Expression and Quantification of Ovis Aries IGF1, IGF2, and mTOR mRNA in Samples of Longissimus Muscle Using Quantitative Real-Time PCR

Two samples of ~0.5 cubic centimeter (cm) from the LM were collected from one lamb/pen (6 lambs/treatment) and snap-frozen in liquid nitrogen, crushed to powder, subdivided in ~100 mg aliquots and stored at −80 °C for total RNA extraction using the Aurum Total RNA Fatty and Fibrous Tissue kit (Bio-Rad, Hercules, CA, USA), following manufacturer’s instructions. Oligonucleotides were designed using the Gene Runner software 6.5 (http://www.generunner.net/ (accessed on 6 March 2023)) and the OligoCalc oligonucleotides properties calculator (http://biotools.nubic.northwestern.edu/OligoCalc.html (accessed on 6 March 2023)) to select DNA fragments with the optimal physical properties to hybridize with specific DNA sequences of Ovis aries IGF1 gene (GenBank: NM_001009774.3), Ovis aries IGF2 (GenBank: NM_001009311.1) and, Ovis aries mTOR gene (GenBank: NM_001145455.1). Tests were performed using a Bio-Rad CFX 96 thermal cycler and optimal PCR conditions were calculated using the Auto Writer tool of the CFX96 Maestro software 2.3 for each set of oligonucleotides and amplicons. A melting curve analysis (Tm) was included in each test run to ensure that individual reactions developed within the expected parameters. Samples were considered positive when the threshold cycle value occurs at ≤38 cycles, the curve in the amplification plot shows an exponential increase and the Tm matches with that of the corresponding DNA reference control. For the quantification of mRNA expression, logarithmic serial dilutions (10^9^ to 10^1^) of each reference DNA control were used to construct a calibration curve. Samples were tested in duplicates and DNA controls in triplicates and compared with the amplification plot to calculate the number of genomic equivalents detected in each sample. Real-time quantitative analysis results were normalized to RPS9 expression levels and relative gene expression was calculated using the 2^−ΔΔCt^ method.

### 2.8. Statistical Analysis

All the data were tested for normality using the Shapiro–Wilk test. Growth performance data (gain, gain efficiency, and dietary energetics), DM intake, and carcass data were analyzed as a randomized complete block design with a 2 × 2 factorial arrangement of treatments, with the pen as the experimental unit, using the MIXED procedures of SAS software 9.3 [29], with treatment and block as fixed effects and the experimental unit within treatment as a random effect. Visceral organ mass and gene expression data were analyzed as a randomized complete block design with a 2 × 2 factorial arrangement of treatments, using the MIXED procedure of SAS software 9.3 [29] with treatment and pen as fixed effects and treatment–pen interaction as random effect. In all cases, contrasts are considered significant when the *p* value < 0.05.

## 3. Results

### 3.1. Ambient Temperature

Ambient temperature and relative humidity (RH) during the experiment are shown in Table 2. Based on temperature and RH, the minimum and maximum calculated average value of THI during the experiment were 79.62 and 81.26, respectively. The average THI (80.4 ± 3.2) was within the range (79–84) coded as “Danger” [30]. Daily maximal THI exceeded 80 for an average of 4.7 h during 52 of the 70 days of the experiment. These ambient conditions are expected to compromise energy intake and, hence, lamb growth performance [31].

### 3.2. Growth Performance and Dietary Energy

There was no morbidity or mortality during the study. Treatment effects on growth performance and dietary net energy are shown in Table 3. There were no treatment interactions. Supplemental EOD3 did not affect (*p* = 0.43) dry matter intake (DMI), but increased (*p* < 0.01) carcass adjusted average daily gain (ADG, 9.2%), gain efficiency (GF, 6.7%), and observed vs. expected dietary NE_m_ and NE_g_ (4.8 and 6.4%, respectively). Supplemental ZH did not affect dry matter intake (DMI, *p* = 0.50) but increased (*p* < 0.01) carcass adjusted ADG (14.5%), GF (13%) and observed vs. expected dietary NE_m_ and NE_g_ (9.0% and 11.7%, respectively). Compared to control lambs, the combination of both additives increased ADG (24.9%), GF (21.2%), and observed vs. expected dietary NE_m_ and NE_g_ (14.2% and 18.9%, respectively).

### 3.3. Carcass Characteristics, Visceral Mass, and Gene Expression for IGF-1, IGF-2 and mTOR in LM Muscle

Treatment effects on carcass characteristics, visceral mass, and gene expression are shown in Table 4, Table 5 and Table 6. There were no treatment interactions on carcass characteristics, visceral organ mass, or on gene expression of IGF1, IGF2 and mTOR in longissimus muscle (LM). Supplemental EOD3 increased hot carcass weight (4.0%, *p* < 0.01) but did not affect the other carcass measures. Supplemental EOD3 decreased (3%, *p* = 0.03) intestine mass weight (g intestine/kg empty body weight). Supplemental ZH increased HCW (6%, *p* < 0.01), dressing percentage (1.7%, *p* = 0.04), and longissimus muscle area (9.7%, *p* < 0.01), and decreased KPH (16.2%, *p* < 0.01), fat thickness (14.7%, *p* = 0.03), and visceral fat. Compared to controls, the combination of EOD3 with ZH increased HCW (10.2%). Supplemental EOD3 did not affect (*p* ≥ 0.38) LM gene expression of IGF1, IGF2 and mTOR. Supplemental ZH did not affect (*p* ≥ 0.29) LM gene expression of IGF2 and mTOR, but increased (92.9%, *p* = 0.03) expression of IGF1.

## 4. Discussion

Conditions of HAHL do not necessarily result in “heat stress”. Several long-term studies noted adaptive changes in dietary intake patterns and energy utilization in cattle fed under HAHL (>79 THI) did not detect changes in “stress parameters” (rectal temperature, blood metabolites, breath rate, etc.) [5]. This adaptation (termed “adaptation physiology”) can have a marked effect on growth performance [32,33]. An objective of this experiment was to determine how the feed additive effects may combine to help to alleviate the negative impact on dietary energy utilization and growth of adapted lambs fattening under high ambient load.

Based on average weight and average DM intake during the experiment, the daily average net intakes of additives were 184, 0.110, and 7.34 mg for EO, D3, and ZH, respectively. These values are equivalent to 5.1, 0.037, 0.20 mg/kg LW. In previous studies, the dose of the same blend of EO used in the present experiment [9,10], as well as the ZH dose ingested and the duration of supplementation [19,20], have shown consistently positive responses on growth performance, dietary energy and/or carcass characteristics when administered separately to finishing Pelibuey lambs. To our knowledge, there is no published research evaluating effects of long term supra-supplementation D3 (administered separately) on growth performance of carcass characteristics in lambs. However, in feedlot cattle, ingestion of 0.003 to 0.007 mg/kg LW enhanced ADG and carcass weight [11,12].

Reduced feed intake, a characteristic response to HAHL, is the primary basis for decreased ADG [32,34]. In the present study, observed DMI for Control and supplemented lambs were in close agreement (1.196 vs. 1.203 and 1.215 kg DM/d, respectively), projected according to NRC [35]. In contrast to non-adapted breeds, elevated ambient temperature has a lesser effect on growth performance of hairy lambs [36,37,38]. Nevertheless, in climatic conditions similar to those recorded in the present study, reductions in DM intake of up to 8% have been reported [9,39]. The absence of an effect of HAHL on DM intake of un-supplemented lambs observed in this experiment is surprising. Although an initial reaction to high environmental temperature is a reduction in DM intake [40], some conditions, including a night-time “cooling” period, help to dissipate some of the excessive heat load. In this experiment, the THI exceeded 80 values in an average of 4.7 h during the day. Therefore, the lambs had a chance to dissipate extra heat load for the rest of the day, thus reducing the negative impact on DM intake. Escobedo-Gallegos et al. [14] observed that EOD3 supplementation (0.12 mg/kg diet) did not prevent depression of DM intake in finishing lambs under HAHL conditions. However, HAHL conditions in that study were more severe, with maximal THI averaging 87.9, and with 6.2 h/d of THI in excess of 80.

The effects of essential oils on DM intake, when EO were supplemented separately, have not been consistent. Most reports did not show an effect of EO on DM intake [41,42,43]. In some studies, EO supplementation increased DM intake [44,45], whereas in others EO supplementation decreased DM intake [46]. These inconsistencies may be attributed to types of essential oil supplemented. The essential oil blend used in the present study (a blend of thymol, eugenol, vanillin, guaiac, and limonene), has not affected DM intake of feedlot cattle or lambs [9,10]. Likewise, supra-supplementation of vitamin D3 (up to 0.007 mg/kg LW) did not affect DM intake [11,47]. Very little has been reported regarding the influence of the combination of essential oils plus vitamin D3 (EOD3) on DM intake in ruminants. In the feedlot cattle, EOD3 supplementation did not affect DM intake when supplemented under HAHL or under favorable climatic conditions [13,48,49]. Consistent with the present study, Escobedo-Gallegos et al. [14] observed that, under HAHL conditions, EOD3 (0.12 mg/kg) did not affect DM intake of finishing lambs. Consistent with the present study, Ortiz et al. [20] in a meta-analysis study observed that ZH supplementation did not appreciable affect DM intake in finishing lambs. The absence of an effect of the combination EOD3 plus ZH on DM intake in the present study is consistent with the above findings.

The impact of HAHL is characterized by reduced feed intake, which is thought to be the primary cause for reduced ADG. There is evidence, however, that HAHL negatively impacts not only feed intake (and therefore energy intake), but also the efficiency of dietary energy utilization [4]. The estimation of dietary energy utilization efficiency using the observed-to-expected ratio, based on the observed growth performance and the diet NE in accordance with tabular values for individual feed ingredients, is a useful and a more precise tool than the conventional measure of “feed efficiency” to express differences in energy utilization for growth performance [27]. An observed-to-expected dietary NE ratio of 1.00 indicates that observed ADG is consistent with that expected, based on DM intake and energy density of the formulated diet. A ratio below 1.00 indicates poor energy utilization for ADG, whereas a value greater than 1.00 indicates more efficient energy than expected. In the current experiment, the DMI was not negatively affected by HAHL, but the efficiency of the energy utilization was lower (7.6%) for Control lambs. This confirms the negative effect of HAHL on the partial efficiency of the energy destined for gain [50]. Put differently, the net energy requirement for maintenance increases, reducing the energy available for weight gain. The magnitude of changes in maintenance requirements for lambs in this experiment can be estimated as described by Estrada-Angulo et al. [51]: Maintenance coefficient (MQ) = (NE_m_ × [DMI − {EG/NE_g_}])/SBW^0.75^], where NE_m_ corresponds to the NE values of the diet (Table 1) according to NRC [23], EG is the energy requirement for gain, and DMI and SBW correspond to the general average values for DM intake and SBW observed during the experiment. Accordingly, HAHL increased the MQ in non-supplemented lambs by 11.8%. This increase in MQ is within range for animals under HAHL conditions specified for NRC [50], and similar to previously reported values (13.0 and 14.7%) for non-supplemented Pelibuey lambs fed similar diets and under similar environmental conditions [14,51]. Lambs supplemented with EOD3 had a 15% decrease in MQ requirement. Lambs supplemented with ZH had an apparent 21% decrease in MQ requirement. Changes in the composition of the tissue gain due to ZH supplementation (promoting more protein and less tissue fat deposition) might have led to the apparent decrease in NE required for maintenance [8,52,53]. Moreover, recent findings indicate that supplemental ZH partially moderated inflammation and oxidative stress skeletal muscle in beef cattle under heat stress [54].

Increases in diet energetic efficiency in ruminants under HAHL condition have been observed in feedlot cattle and lambs. Escobedo-Gallegos et al. [14] observed a 3.7% increase in diet energetic efficiency in Pelibuey Katahdin lambs supplemented with EOD3 under HAHL conditions. Likewise, EOD3 supplementation of feedlot cattle under HAHL conditions (average THI = 82.7) increased dietary energetic efficiency by 3.0% compared to cattle that were supplemented with the ionophore monensin. The basis for improvements in efficiency of net energy utilization may be attributable to changes in ruminal fermentation, intestinal epithelial cells, and cellular oxidative status [55], although other studies also indicate that EOD3 supplementation under HAHL conditions alleviated heat load by lowering intra-ruminal and rectal temperature [48,56]. The improvement of energy efficiency mediated by ZH supplementation observed here could be explained mainly by its repartitioning agent effect, even under HAHL conditions [8]. The effects of the combination of EOD3 with ZH on energetic efficiency were additive. There are no previous reports that directly evaluate the combination of these additives.

Although EO supplementation enhances growth performance, it did not affect carcass measures [10,57]. Supra-supplementation with vitamin D3 resulted in increased carcass weight and dressing percentage [11,12]. This may be attributable to increased expression of genes affecting muscle growth and protein synthesis [58,59]. However, in the present study there were no appreciable effects of D3 in combination with EO on carcass measures. In feedlot cattle fed a corn-based finishing diet (2.14 Mcal NE_m_/kg diet), supplementation with EOD3 (120 mg EO+0.12 mgD3/kg diet) increased LM area, but did not affect other carcass measures [49]. In Holstein steers fed a steam flaked corn-based diet (2.20 Mcal NE_m_/kg) during a 285 d growing-finishing period, supplementation with EOD3 (200 mg/kg diet) reduced carcass fat thickness without effects on other carcass characteristics [48]. In feedlot lambs finished under environmental conditions similar to those of the present study, EOD3 supplementation decreased internal fat (KPH) without effects on other carcass measures [14].

We did not detect an effect of D3 supplemented in combination with EO on measures of LM muscle gene expression. Muscle tissue concentration of IGFs and mTOR are associated with anabolic and catabolic signaling of skeletal muscle, affecting the modulation of muscle hypertrophy [60]. Thus, increases or decreases in these metabolites are expected to impact on some carcass traits. Martin et al. [58] observed a tendency for increased gene expression of IGF1, IGF2, MTOR in muscle of feedlot cattle supplemented with 1 mg D3/d (approximately 0.0022 mg/kg BW) during an 85 d feedlot phase.

As observed in the present study, increases in hot carcass weight, dressing percentage, and LM area, with reductions in fat thickness and KPH, are consistent responses to ZH supplementation [19,20,61]. However, several factors may influence the magnitude of responses when ZH is supplemented, including dose [7,62], supplementation period [63], withdrawal period [64], gender [65], slaughter weight [66], and ZH type (patented or generic [19]. Enhancement in carcass characteristics in the present study may be attributable to increased LM muscle IGF1 expression, and ZH supplementation did not affect IGF2 and mTOR expression.

Reduction in the relative proportion (g/kg EBW) of intestinal mass as a result of EO supplementation has been observed previously [9,10,67]. Although the mechanism is not clear, it could be related to an antibiotic-like effect on epithelial thickness [68]. The effect of ZH supplementation on visceral mass is associated with a general decrease in visceral fat [69,70]. The combination of EOD3 with ZH did not alter these effects.

## 5. Conclusions

It is concluded that growth performance responses to supplemental EOD3 and ZH are additive. Both supplements can be fed in combination without detrimental effects on expected benefits to ADG, gain efficiency, efficiency of energy utilization and carcass measures when fed separately. Both, EOD3 and ZH, supplemented separately or combined, can be used as a strategy to improve gain efficiency and dietary energy utilization in lambs fattening under high-ambient-load.

## Figures and Tables

**Table 1:** Composition of basal diet and treatments.

	Treatments ^1^			
Item	Control	EOD3	ZH	EOD3 + ZH
Ingredient composition (%)				
Sudangrass hay	10.00	10.00	10.00	10.00
Cracked corn	68.00	68.00	68.00	68.00
Soybean meal	10.00	10.00	10.00	10.00
CRINA + HyD	---	+++	---	+++
ZH	---	---	+++	+++
Molasses cane	6.00	6.00	6.00	6.00
Yellow grease	2.50	2.50	2.50	2.50
Zeolite clay	1.00	1.00	1.00	1.00
Trace protein-mineral salt ^2^	2.50	2.50	2.50	2.50
Chemical composition (%DM basis) ^3^				
Crude protein	14.20	14.20	14.20	14.20
Neutral detergent fiber	15.43	15.43	15.43	15.43
Dry matter	88.32	88.32	88.32	88.32
Calculated net energy (Mcal/kg) ^4^				
Maintenance	2.10	2.10	2.10	2.10
Gain	1.44	1.44	1.44	1.44

The symbol “+++” indicate supplementation, the symbol “---“ indicate without supplementation. ^1^ Control = basal diet without feed additives; EOD3 = 150 mg of essential oils blend plus 0.09 mg of 25-hydroxy-Vit-D3/kg of diet offered during all experimental period; ZH = basal diet during the first 35 days and zilpaterol hydrochloride supplementation at 6.0 mg/kg of diet offered during the final 35 days of the experiment (33 days with ZH with a withdrawal 3-d at moment of harvest, and EOD3 + ZH = basal diet supplemented with EOD3 during first 35 days and finishing with the combination with ZH (**EOD3 + ZH**) during the final 35 days of the experiment with a 3-d withdrawal from ZH before harvest. ^2^ Mineral premix contained: Crude protein 72.8%, Calcium, 20%; CoSO_4_, 0.010%; CuSO_4_, 0.15%; FeSO_4_, 0.528%; ZnO, 0.111%; MnSO_4_, 0.160%; KI, 0.007%; and NaCl, 13.7%. ^3^ Crude protein, neural detergent fiber and dry matter were determined in our laboratory. ^4^ Net energy was calculated based on tabular net energy (NE) values for individual feed ingredients [23].

**Table 2:** Ambient temperature (Ta), relative humidity (RH) and calculated temperature–humidity index (THI) ^1^ registered every hour and expressed as a weekly average.

Week	Mean T_a_ (°C)	Max T_a_ (°C)	Min T_a_ (°C)	Mean RH (%)	Max RH (%)	Min RH (%)	Mean THI ^1^	Max THI	Min THI
1	30.18 ± 3.8	30.54 ± 3.9	29.83 ± 3.6	72.73 ± 12.9	74.18 ± 12.4	70.99 ± 13.4	81.93 ± 3.9	82.74 ± 4.2	81.14 ± 3.5
2	28.42 ± 2.9	28.80 ± 3.0	28.04 ± 2.7	80.58 ± 11.3	82.18 ± 10.7	79.01 ± 11.8	80.47 ± 3.0	81.33 ± 3.4	79.67 ± 2.7
3	29.78 ± 3.1	30.10 ± 3.2	29.47 ± 3.0	75.04 ± 12.6	76.60 ± 12.1	73.58 ± 12.9	81.79 ± 3.1	82.55 ± 3.3	81.09 ± 3.0
4	30.21 ± 2.4	30.51 ± 2.6	29.88 ± 2.3	73.44 ± 9.7	74.87 ± 9.5	72.12 ± 9.7	82.33 ± 2.5	83.04 ± 2.8	81.64 ± 2.3
5	30.00 ± 3.3	30.38 ± 3.4	29.66 ± 3.2	72.23 ± 13.3	73.75 ± 13.0	70.61 ± 13.5	81.63 ± 3.3	82.47 ± 3.5	80.88 ± 3.1
6	29.20 ± 3.4	29.56 ± 3.4	28.80 ± 3.3	70.10 ± 16.0	72.02 ± 15.4	68.36 ± 16.4	79.96 ± 2.8	80.82 ± 3.1	79.11 ± 2.5
7	30.77 ± 3.8	31.18 ± 3.8	30.33 ± 3.7	68.34 ± 15.5	70.44 ± 15.2	66.38 ± 15.6	82.01 ± 3.1	83.00 ± 3.3	81.06 ± 3.0
8	27.46 ± 3.5	27.81 ± 3.6	27.12 ± 3.4	76.10 ± 15.5	77.62 ± 15.0	74.53 ± 16.0	78.13 ± 3.2	78.88 ± 3.5	77.40 ± 3.0
9	28.39 ± 3.8	28.80 ± 3.9	27.99 ± 3.8	72.84 ± 16.9	74.53 ± 16.5	71.17 ± 17.2	79.02 ± 3.4	79.89 ± 3.7	78.18 ± 3.3
10	27.29 ± 4.1	27.73 ± 4.1	26.86 ± 3.9	69.97 ± 17.0	71.70 ± 16.6	68.18 ± 17.0	76.94 ± 3.9	77.84 ± 4.1	76.08 ± 3.7
Mean	29.17 ± 3.4	29.54 ± 3.5	28.80 ± 3.3	73.14 ± 14.1	74.79 ± 13.6	71.49 ± 14.3	80.42 ± 3.2	81.26 ± 3.5	79.62 ± 3.0

^1^ THI = 0.81 × ambient temperature + ((relative humidity × (ambient temperature–14.4)) + 46.4. THI code (normal THI < 74; alert 75 to 79; danger 79 to 84; and emergency > 84).

**Table 3:** Treatment effects on growth performance and dietary energy during fattening period (70 d) ^1^.

	−ZH		+ZH		EOD3, mg/kg DM			ZH, mg/kg DM			EOD3 × ZH	
Item	−EOD3	+EOD3	−EOD3	+EOD3	0	151	*p* Value	0	6	*p* Value	*p* Value	SEM
Pen replicates	6	6	6	6	12	12		12	12			
Live weight, kg												
Initial	25.23	25.15	25.07	25.02	25.15	25.08	0.55	25.19	25.05	0.23	0.92	0.116
Final ^2^	43.75	45.06	45.97	48.22	44.86	46.64	<0.01	44.41	47.09	<0.01	0.29	0.307
Weight gain, kg/d	0.265	0.285	0.299	0.331	0.282	0.308	<0.01	0.275	0.315	<0.01	0.30	0.008
DM intake, kg	1.203	1.199	1.195	1.251	1.199	1.225	0.43	1.201	1.223	0.50	0.37	0.031
Gain to feed ratio	0.221	0.238	0.252	0.268	0.237	0.253	<0.01	0.230	0.260	<0.01	0.92	0.003
Diet energy, Mcal/kg												
Maintenance	1.958	2.064	2.147	2.238	2.053	2.152	<0.01	2.011	2.193	<0.01	0.79	0.029
Gain	1.307	1.404	1.473	1.553	1.390	1.477	<0.01	1.354	1.513	<0.01	0.79	0.026
Observed-to-expected diet NE												
Maintenance	0.934	0.984	1.024	1.067	0.979	1.026	<0.01	0.959	1.046	<0.01	0.79	0.014
Gain	0.914	0.980	1.031	1.087	0.972	1.034	<0.01	0.947	1.059	<0.01	0.79	0.013
Observed-to-expected DM intake	1.082	1.016	0.971	0.921	1.026	0.969	<0.01	1.049	0.946	<0.01	0.64	0.012

^1^ EOD3 = 150 mg of essential oils blend plus 0.09 mg of 25-hydroxy- Vit-D3/kg of diet offered during all experimental period; ZH = basal diet during the first 35 days and zilpaterol hydrochloride supplementation at 6.0 mg/kg of diet offered during the final 35 days of the experiment (33 days with ZH with a withdrawal 3-d at moment of harvest, and EOD3 + ZH = basal diet supplemented with EOD3 during first 35 days and finishing with the combination with ZH (**EOD3 + ZH**) during the final 35 days of the experiment with a 3-d withdrawal from ZH before harvest. ^2^ Because there was a significant difference in carcass dressing percentage between the treatments, final SBW was adjusted for hot carcass weight (HCW) by dividing individual HCW by the average dressing percentage (0.5813) for all lambs.

**Table 4:** Treatment effects on carcass characteristics and tissue composition in lambs ^1^.

	−ZH		+ZH		EOD3, mg/kg DM			ZH, mg/kg DM			EOD3 × ZH	
Item	−EOD3	+EOD3	−EOD3	+EOD3	0	151	*p* Value	0	6	*p* Value	*p* Value	SEM
Lamb replicates	6	6	6	6	12	12		12	12			
HCW, kg	25.43	26.20	26.73	28.03	26.07	27.12	<0.01	25.81	27.37	<0.01	0.29	0.252
Dressing percentage	57.20	58.08	58.52	58.73	57.86	58.41	0.24	57.64	58.62	0.04	0.47	0.210
CCW, kg	25.07	25.93	26.30	27.70	25.69	26.81	<0.01	25.50	27.07	<0.01	0.29	0.249
LM area, cm^2^	15.02	15.45	16.85	16.57	16.44	16.11	0.28	15.23	16.71	0.01	0.41	0187
Fat thickness, cm	0.307	0.320	0.252	0.282	0.279	0.300	0.27	0.313	0.267	0.03	0.67	0.019
KPH, %	2.98	3.20	2.46	2.72	2.72	2.96	0.63	3.095	2.59	<0.01	0.07	0.120

^1^ EOD3 = 150 mg of essential oils blend plus 0.09 mg of 25-hydroxy- Vit-D3/kg of diet offered during all experimental period; ZH = basal diet during the first 35 days and zilpaterol hydrochloride supplementation at 6.0 mg/kg of diet offered during the final 35 days of the experiment (33 days with ZH with a withdrawal 3-d at moment of harvest, and EOD3 + ZH = basal diet supplemented with EOD3 during first 35 days and finishing with the combination with ZH (**EOD3 + ZH**) during the final 35 days of the experiment with a 3-d withdrawal from ZH before harvest. HCW = hot carcass weight. CCW = cold carcass weight. LM = longissimus muscle.

**Table 5:** Treatment effects on visceral mass in lambs ^1^.

	−ZH		+ZH		EOD_3_, mg/kg DM			ZH, mg/kg DM			EOD_3_ × ZH	
Item	−EOD_3_	+EOD_3_	−EOD_3_	+EOD_3_	0	151	*p* Value	0	6	*p* Value	*p* Value	SEM
Pen replicates	6	6	6	6	12	12		12	12			
EBW, % of full weight	90.57	90.43	91.08	90.47	90.57	91.08	0.43	90.43	90.47	0.56	0.61	0.464
Organs, g/kg EBW												
Stomach complex	25.26	25.75	23.97	24.34	24.61	25.04	0.63	25.51	24.15	0.15	0.94	0.877
Intestines	39.75	35.68	39.60	38.29	39.67	36.98	0.03	37.72	38.94	0.29	0.24	1.140
Liver	17.67	18.25	16.21	17.34	16.94	17.80	0.31	17.96	16.77	0.17	0.74	0.809
Hearth + lungs	19.81	21.07	19.49	20.56	19.65	20.81	0.25	20.44	20.02	0.67	0.92	0.963
Kidney	2.97	2.99	2.92	2.91	2.94	2.95	0.98	2.98	2.91	0.73	0.91	0.178
Visceral fat	32.09	33.92	27.42	27.98	29.75	30.95	0.61	33.00	27.70	0.04	0.78	1.629

^1^ EOD3 = 150 mg of essential oils blend plus 0.09 mg of 25-hydroxy- Vit-D3/kg of diet offered during all experimental period; ZH = basal diet during the first 35 days and zilpaterol hydrochloride supplementation at 6.0 mg/kg of diet offered during the final 35 days of the experiment (33 days with ZH with a withdrawal 3-d at moment of harvest, and EOD3 + ZH = basal diet supplemented with EOD3 during first 35 days and finishing with the combination with ZH (**EOD3 + ZH**) during the final 35 days of the experiment with a 3-d withdrawal from ZH before harvest).

**Table 6:** Treatment effects on IGF-1, IGF-2 and mTOR RNA genomic equivalents in LM muscle in lambs ^1^.

	−ZH		+ZH		EOD_3_, mg/kg DM			ZH, mg/kg DM			EOD_3_ × ZH	
Item	−EOD_3_	+EOD_3_	−EOD_3_	+EOD_3_	0	151	*p* Value	0	6	*p* Value	*p* Value	SEM
Pen replicates	6	6	6	6	12	12		12	12			
IGF-1	7.92	8.93	18.93	13.54	13.42	11.24	0.47	8.42	16.23	0.03	0.49	2.61
IGF-2	8.85	9.93	12.31	10.49	10.58	10.44	0.64	9.39	11.40	0.29	0.88	1.64
mTOR	1.85	1.89	1.84	1.95	1.85	1.92	0.38	1.87	1.88	0.74	0.94	0.076

^1^ EOD3 = 150 mg of essential oils blend plus 0.09 mg of 25-hydroxy- Vit-D3/kg of diet offered during all experimental period; ZH = basal diet during the first 35 days and zilpaterol hydrochloride supplementation at 6.0 mg/kg of diet offered during the final 35 days of the experiment (33 days with ZH with a withdrawal 3-d at moment of harvest, and EOD3 + ZH = basal diet supplemented with EOD3 during first 35 days and finishing with the combination with ZH (**EOD3 + ZH**) during the final 35 days of the experiment with a 3-d withdrawal from ZH before harvest).

## Data Availability

The data that support the findings of this study are available from the corresponding authors upon reasonable request.

## References

[1] Morris S.T., Ferguson D.M., Lee C., Fisher A. (2017). Overview of Sheep Production Systems. Advances If Sheep Welfare.

[2] Hahn G.L. (1999). Dynamic responses of cattle to thermal heat loads. J. Anim. Sci..

[3] Osei-Amponsah R., Chauhan S.S., Leury B.J., Cheng L., Cullen B., Clarke I.J., Dunshea F.R. (2019). Genetic Selection for Thermotolerance in Ruminants. Animals.

[4] Chauhan S.S., Rashamol V.P., Bagath M., Sejian V., Dunshea F.R. (2021). Impacts of heat stress on immune responses and oxidative stress in farm animals and nutritional strategies for amelioration. Int. J. Biometeorol..

[5] Renaudeau D., Collin A., Yahav S., de Basilio V., Gourdine J.L., Collier R.J. (2012). Adaptation to hot climate and strategies to alleviate heat stress in livestock production. Animals.

[6] Castro-Pérez B.I., Estrada-Angulo A., Ríos-Rincón F.G., Núñez-Benítez V.H., Rivera-Méndez C.R., Urías-Estrada J.D., Zinn R.A., Barreras A., Plascencia A. (2020). The influence of shade allocation or total shade plus overhead fan on growth performance, efficiency of dietary energy utilization, and carcass characteristics of feedlot cattle under tropical ambient conditions. Asian-Australas. J. Anim. Sci..

[7] Cayetano-De-Jesus J.A., Rojo-Rubio R., Grajales-Lagunes A., Avendaño-Reyes L., Macias-Cruz U., Gonzalez-del-Prado V., Olmedo-Juárez A., Chay-Canul A., Roque-Jiménez J.A., Lee-Rangel H.A. (2020). Effect of zilpaterol hydrochloride on performance and meat quality in finishing lambs. Agriculture.

[8] Barnes T.L., Cadaret C.N., Beede K.A., Schmidt T.B., Petersen J.L., Yates D.T. (2019). Hypertrophic muscle growth and metabolic efficiency were impaired by chronic heat stress, improved by zilpaterol supplementation, and not affected by ractopamine supplementation in feedlot lambs. J. Anim. Sci..

[9] Arteaga-Wences Y., Estrada-Angulo A., Gerardo Ríos-Rincón F.G., Castro-Pérez B.I., Mendoza-Cortéz D.A., Manriquez-Núñez O.M., Barreras A., Corona-Gochi L., Zinn R.A., Perea-Domínguez X.P. (2021). The effects of feeding a standardized mixture of essential oils vs. monensin on growth performance, dietary energy and carcass characteristics of lambs fed a high-energy finishing diet. Small Rum. Res..

[10] Estrada-Angulo A., Arteaga-Wences Y.J., Castro-Pérez B.I., Urías-Estrada J.D., Gaxiola-Camacho S., Angulo-Montoya C., Ponce-Barraza E., Barreras A., Corona L., Zinn R.A. (2021). Blend of Essential Oils Supplemented Alone or Combined with Exogenous Amylase Compared with Virginiamycin Supplementation on Finishing Lambs. Animals.

[11] Acedo T.S., Gouvêa V.N., Vasconcellos G.M., Arrigoni M., Martins C.L., Millen D.D., Muller L.R., Melo G.F., Rizzieri R.A., Costa C.F. (2019). Effect of 25-hydroxy-vitamin-D3 on feedlot cattle. J. Anim. Sci..

[12] Carvalho V.V., Perdigão A. (2019). Supplementation of 25-hydroxy-vitamin-D3 and increased vitamin E as a strategy to increase carcass weight of feedlot beef cattle. J. Anim. Sci..

[13] Mendoza-Cortéz D.A., Ramos-Méndez J.L., Arteaga-Wences Y., Félix-Bernal A., Estrada-Angulo A., Castro-Pérez B.I., Urías-Estrada J.D., Barreras A., Zinn R.A., Plascencia A. (2022). Influence of a supplemental blend of essential oils plus 25-hydroxy-vitamin-d3 on feedlot cattle performance during the early-growing phase under conditions of high-ambient temperature. Indian J. Anim. Res..

[14] Escobedo-Gallegos L.d.G., Estrada-Angulo A., Castro-Pérez B.I., Urías-Estrada J.D., Calderón-Garay E., Ramírez-Santiago L., Valdés-García Y.S., Barreras A., Zinn R.A., Plascencia A. (2023). Essential Oils Combined with Vitamin D3 or with Probiotic as an Alternative to the Ionophore Monensin Supplemented in High-Energy Diets for Lambs Long-Term Finished under Subtropical Climate. Animals.

[15] Normas Oficiales Mexicanas (1995). Diario Oficial de la Federación. (NOM-051-ZOO-1995, NOM-033-ZOO-1995) Trato Humanitario de Animales de Producción, de Compañía y Animales Silvestres Durante el Proceso de Crianza, Desarrollo de Experimentos, Movilización y Sacrificio. https://dof.gob.mx/nota_detalle.php?codigo=4870842&fecha=23/03/1998#gsc.tab=0.

[16] Dikmen S., Hansen P.J. (2009). Is the temperature-humidity index the best indicator of heat stress in lactating dairy cows in a subtropical environment?. J. Dairy Sci..

[17] Rojas-Román L.A., Castro-Pérez B.I., Estrada-Angulo A., Angulo-Montoya C., Yocupicio-Rocha J.A., López-Soto M.A., Barreras A., Zinn R.A., Plascencia A. (2017). Influence of long-term supplementation of tannins on growth performance, dietary net energy and carcass characteristics in finishing lambs. Small Rum. Res..

[18] Estrada-Angulo A., Castro-Pérez B.I., Urías-Estrada J.D., Ríos-Rincón F.G., Arteaga-Wences Y.J., Barreras A., López-Soto M.A., Plascencia A., Zinn R.A. (2018). Influence of protein level on growth performance, dietary energetics and carcass characteristics of Pelibuey × Katahdin lambs finished with isocaloric diets. Small Rum. Res..

[19] Rivera-Villegas A., Estrada-Angulo A., Castro-Pérez B.I., Urías-Estrada J.D., Ríos-Rincón F.G., Rodríguez-Cordero D., Barreras A., Plascencia A., González-Vizcarra V.M., Sosa-Gordillo J.F. (2019). Comparative evaluation of supplemental zilpaterol hydrochloride sources on growth performance, dietary energetics and carcass characteristics of finishing lambs. Asian-Australas. J. Anim. Sci..

[20] Ortiz-Rodea A., Barbosa-Amezcua M., Partida J.A., González-Ronquillo M. (2016). Effect of zilpaterol hydrochloride on animal performance and carcass characteristics in sheep: A meta-analysis. J. Appl. Anim. Res..

[21] Cannas A., Tedeschi L.O., Fox D.G., Pell A.N., Van Soest P.J. (2004). A mechanistic model for predicting the nutrient requirements and feed biological values for sheep. J. Anim. Sci..

[22] Association of Official Analytical Chemists (2000). Official Method of Analysis.

[23] National Research Council (2007). Nutrient Requirement of Small Ruminant: Sheep, Goats, Cervids, and New World Camelids.

[24] Van Soest P.J., Robertson J.B., Lewis B.A. (1991). Methods for Dietary Fiber, Neutral Detergent Fiber, and Nonstarch Polysaccharides in Relation to Animal Nutrition. J. Dairy Sci..

[25] National Research Council (1985). Nutrient Requirement of Sheep.

[26] Canton G.J., Bores Q.R., Baeza R.J., Quintal F.J., Santos R.R., Sandoval C.C. (2009). Growth and Feed efficiency of Pure and F1 Pelibuey Lambs Crossbred with Specialized Breeds for Production of Meat. J. Anim. Vet. Adv..

[27] Zinn R.A., Barreras A., Owens F.N., Plascencia A. (2008). Performance by feedlot steers and heifers: ADG, mature weight, DMI and dietary energetics. J. Anim. Sci..

[28] Agricultural Marketing Service (USDA) (1992). Official United States Standards for Grades of Carcass Lambs, Yearling Mutton and Mutton Carcasses.

[29] Statistical Analytical System, Institute Inc (2004). SAS Proprietary software Release 9.3.

[30] Mader T.L., Davis M.S., Brown-Brandl T. (2006). Environmental factors influencing heat stress in feedlot cattle. J. Anim. Sci..

[31] Silanikove N. (2000). Effects of heat stress on the welfare of extensively managed domestic ruminants: A review. Livest. Prod. Sci..

[32] Lees A.M., Sejian V., Wallage A.L., Steel C.C., Mader T.L., Lees J.C., Gaughan J.B. (2019). The Impact of Heat Load on Cattle. Animals.

[33] Moreno J.A., Lopes de Sá A., Cohelo C.F., Pereira R.N., Batista E.D., Ladeira M.M., Casagrande D.R., Gionbelli M.P. (2021). Effect of heat stress on ingestive, digestive, ruminal and physiological parameters of Nellore cattle feeding low- or high-energy diets. Livest. Sci..

[34] Mahjoubi E., Yazdi M.H., Aghaziarati N., Noori G.R., Afsarian O., Baumgard L.H. (2015). The effect of cyclical and severe heat stress on growth performance and metabolism in Afshari lambs. J. Anim. Sci..

[35] National Research Council (1987). Predicting Feed Intake of Producing Animals.

[36] Vicente-Pérez V.R., Macías-Cruz U., Avendaño-Reyes L., Correa-Calderón A., López-Vaca M.A., Lara-Rivera A.L. (2020). Heat stress impacts in hair sheep production. Rev. Mex. Cienc. Pecu..

[37] Macías-Cruz U., Saavedra O.R., Correa-Calderón A., Mellado M., Torrentera N.G., Chay-Canul A., López-Baca M.A., Avendaño-Reyes L. (2020). Feedlot growth, carcass characteristics and meat quality of hair breed lambs exposed to seasonal heat stress (winter vs. summer) in an arid climate. Meat Sci..

[38] Nicolás-López P., Macías-Cruz U., Mellado M., Correa-Calderón A., Meza-Herrera C.A., Avendaño-Reyes L. (2021). Growth performance and changes in physiological, metabolic and hematological parameters due to outdoor heat stress in hair breed male lambs finished in feedlot. Int. J. Biometeorol..

[39] Macías-Cruz J., López-Baca M.A., Vicente R., Mejía A., Álvarez F.D., Correa-Calderón A. (2015). Effects of seasonal ambient heat stress (spring vs. summer) on physiological and metabolic variables in hair sheep located in an arid region. Int. J. Biometeorol..

[40] Habebb A.A., Gad A.E., Atta M.A. (2018). Temperature-humidity index as indicators to stress of climatic conditions with relation to production and reproduction of farm animals. Int. J. Biotechnol. Res. Adv..

[41] Beenchar C., Calsamiglia S., Chaves A.V., Fraser G.R., Colombatto D., McCallister T.A., Beauchemin K.A. (2008). A review of plant-derived essential oils in ruminant nutrition and production. Anim. Sci. Technol..

[42] Chaves A.V., Stanford K., Gibson L., McAllister T.A., Benchaar C. (2008). Effects of cinnamaldehyde, garlic and juniper berry essential oils on rumen fermentation, blood metabolites, growth performance, and carcass characteristics of growing lambs. J. Drug Deliv. Sci. Technol..

[43] Moura L.V., Oliveira E.R., Fernandes A.R.M., Gabriel A.M.A., Silva L.H.X., Tkiya C.S., Consolo N.R.B., Rodrigues G.C., Lemos T., Gandra J.R. (2017). Feed efficiency and carcass traits of feedlot lambs supplemented either monensin or increasing doses of copaiba (*Copaifera* spp.) essential oil. Anim. Feed Sci. Technol..

[44] de Souza K.A., Monsteschio J.O., Mottin C., Ramos T.R., Pinto L.A.M., Eiras C.E., Guerrero A., Prado I.N. (2019). Effects of diet supplementation with clove and rosemary essential oils and protected oils (eugenol, thymol and vanillin) on animal performance, carcass characteristics, digestibility, and ingestive behavior activities for Nellore heifers finished in feedlot. Livest. Sci..

[45] Dorantes-Iturbide G., Orzuna-Orzuna J.F., Lara-Bueno A., Mendoza-Martínez G.D., Miranda-Romero L.A., Lee-Rangel H.A. (2022). Essential Oils as a Dietary Additive for Small Ruminants: A Meta-Analysis on Performance, Rumen Parameters, Serum Metabolites, and Product Quality. Vet. Sci..

[46] Parvar R., Ghoorchi T., Kashfi H., Parvar K. (2018). Effect of Ferulago angulata (Chavil) essential oil supplementation on lamb growth performance and meat quality characteristics. Small Rum. Res..

[47] Wang L.H., Zhang C.R., Zhang Q.Y., Xu H.J., Feng G.Z., Zhang G.N., Zhang Y.G. (2022). Effects of feeding different doses of 25-hydroxyvitamin D3 on the growth performance, blood minerals, antioxidant status and immunoglobulin of preweaning calves. Anim. Feed Sci. Technol..

[48] Latack B.C., Carvalho P.H.V., Zinn R.A. (2022). The interaction of feeding an eubiotic blend of essential oils plus 25-hydroxy-vit-D3 on performance, carcass characteristics, and dietary energetics of calf-fed Holstein steers. Front. Vet. Sci..

[49] Estrada-Angulo A., Mendoza-Cortéz D.A., Ramos-Méndez J.L., Arteaga-Wences Y., Urías-Estrada J.D., Castro-Pérez B.I., Ríos-Rincón F.G., Rodríguez-Gaxiola M.A., Barreras A., Zinn R.A. (2022). Comparing Blend of Essential Oils Plus 25-Hydroxy-Vit-D3 Versus Monensin Plus Virginiamycin Combination in Finishing Feedlot Cattle: Growth Performance, Dietary Energetics, and Carcass Traits. Animals.

[50] National Research Council (1981). Effect of Environment on Nutrient Requirements of Domestic Animals (NRC).

[51] Estrada-Angulo A., Aguilar-Hernández A., Osuna-Pérez M., Núñez-Benítez V.H., Castro-Pérez B.I., Silva-Hidalgo G., Contreras-Pérez G., Barreras A., Plascencia A., Zinn R.A. (2016). Influence of quaternary benzophenantridine and protopine alkaloids on growth performance, dietary energy, carcass traits, visceral mass and rumen health in finishing ewes under conditions of severe temperature humidity index. Asian-Australas. J. Anim. Sci..

[52] Moody D.E., Hancock D.L., Anderson D.B., D’Mello J.P.F. (2000). Phenylethanolamine repartitioning agents. Farm Animal Nutrition.

[53] Johnson B.L., Chung K.Y., Hollis L.C., Olson K.C. (2007). Alteration in the physiology of growth cattle with growth-enhancing compounds. Veterinary Clinics of North America: Food Animal Practice.

[54] Reith R.R., Sieck R.L., Grijalva P.C., Swanson R.M., Fuller A.M., Díaz D.E., Schmidt T.B., Yates D.T., Petersen J.L. (2022). Transcriptome analyses indicate that heat stress-induced inflammation in white adipose tissue and oxidative stress in skeletal muscle is partially moderated by zilpaterol supplementation in beef cattle. J. Anim. Sci..

[55] Nehme R., Andrés S., Pereira R.B., Ben Jemaa M., Bouhallab S., Ceciliani F., López S., Rahali F.Z., Ksouri R., Pereira D.M. (2021). Essential Oils in Livestock: From Health to Food Quality. Antioxidants.

[56] Brito da Silva R., Pereira M.N., Canonenco de Araujo R., Silva W.R., Pereira R.A.N. (2020). A blend of essential oils improved feed efficiency and affected ruminal and systemic variables of dairy cows. Transl. Anim. Sci..

[57] Toseti L.B., Goulart R.S., Gouvêa V.N., Acedo T.S., Guillerme S.F.M., Pires A.V., Leme P.R., Netto A.S., Silva S.L. (2020). Effects of a blend essential oils and exogenous amylase in diets containing different roughage sources for finishing beef cattle. Anim. Feed Sci. Technol..

[58] Martins T.E., Acedo T.S., Gouvêa V.N., Vasconcellos G.M., Arrigoni M., Martins C.L., Millen D.D., Pai M.D., Perdigão A., Melo G.F. (2020). Effects of 25-hydroxycholecalciferol supplementation on gene expression of feedlot cattle. J. Anim. Sci..

[59] Romeu-Montenegro K., Pufal M.M., Newsholme P. (2021). Vitamin D Supplementation and Impact on Skeletal Muscle Function in Cell and Animal Models and an Aging Population: What Do We Know So Far?. Nutrients.

[60] Yoon M.S. (2017). mTOR as a key regulator in maintaining skeletal muscle mass. Front. Physiol..

[61] Partida A., Casaya T.A., Rubio M.S., Méndez R.D. (2015). Effect of zilpaterol hydrochloride on the carcass characteristics of Katahdin lamb terminal crosses. Vet. Mex. OA.

[62] Estrada-Angulo A., Barreras-Serrano A., Contreras G., Obregon J.F., Robles-Estrada J.C., Plascencia A., Zinn R.A. (2008). Influence of level of zilpaterol chlorhydrate supplementation on growth performance and carcass characteristics of feedlot lambs. Small Rum. Res..

[63] Vahedi V., Towhidi A., Hedayat-Evrigh N., Vaseghi-Dodaran H., Motlagh M., Ponnampalam E.N. (2015). The effects of supplementation methods and length of feeding of zilpaterol hydrochloride on meat characteristics of fattening lambs. Small Rum. Res..

[64] Robles-Estrada J.C., Arrizon A., Calderon-Cortés J.F., Figueroa-Saavedra F., Torrentera N., Plascencia A., Zinn R.A. (2009). Effects of pre-harvest withdrawal period on response of feedlot heifers to zilpaterol hydrochloride supplementation: Growth performance and carcass characteristics. J. Anim. Sci..

[65] Montgomery J.L., Krehbiel C.R., Cranston J.J., Yates D.A., Hutcheson J.P., Nichols W.T., Streeter M.N., Swingle R.S., Montgomery T.H. (2009). Effects of dietary zilpaterol hydrochloride on feedlot performance and carcass characteristics of beef steers fed with and without monensin and tylosin. J. Anim. Sci..

[66] Castro-Pérez B.I., Estrada-Angulo A., Urías-Estrada J.D., Lazalde-Cruz R., Barreras A., Figueroa-Saavedra F., Zinn R.A., Plascencia A. (2021). Effects of duration of zilpaterol supplementation on growth performance, dietary energetics, carcass characteristics, and meat quality in crossbred yearling heifers when harvested at lighter, less than mature final weight (460 kg). Appl. Anim. Sci..

[67] Wang H., Liang S., Li X., Yang X., Long F., Yang X. (2019). Effects of encapsulated essential oils and organic acids on laying performance, egg quality, intestinal morphology, barrier function, and microflora count of hens during the early laying period. Poult. Sci..

[68] Ghazanfari S., Mohammadi Z., Adib-Moradi M. (2015). Effects of coriander essential oil on the performance, blood characteristics, intestinal microbiota and histological of broilers. Braz. J. Poult. Sci..

[69] Ríos-Rincón F.G., Barreras-Serrano A., Estrada-Angulo A., Obregón J.F., Plascencia-Jorquera A., Portillo-Loera J.J., Zinn R.A. (2010). Effect of level of dietary zilpaterol hydrochloride (β2-agonist) on performance, carcass characteristics and visceral organ mass in hairy lambs fed all-concentrate diets. J. Appl. Anim. Res..

[70] Masoumi R., Afsharirad A., Mirzaei-Alamouti H., Vahedi V., Green M., Aliyari D. (2021). Does fat-tail docking and Zilpaterol hydrochloride (ZH) supplementation affect feedlot performance and carcass characteristics of finishing lambs?. Small Rum. Res..

